# Two New Families and a Literature Review of *ELOVL4-*Associated Spinocerebellar Ataxia Type 34

**DOI:** 10.1007/s12311-023-01522-8

**Published:** 2023-01-25

**Authors:** Masahiro Nishide, Kathleen Le Marquand, Mark R. Davis, Gábor M. Halmágyi, Avi Fellner, Ramesh K. Narayanan, Marina L. Kennerson, Stephen W. Reddel, Lisa Worgan, Peter K. Panegyres, Kishore R. Kumar

**Affiliations:** 1 Sydney Medical School, University of Sydney, Camperdown, NSW 2050 Australia; 2 Clinical Genetics Service, Royal Prince Alfred Hospital, Camperdown, NSW 2050 Australia; 3 Department of Diagnostic Genomics, Path West Laboratory Medicine, QEII Medical Centre, Hospital Avenue, Nedlands, WA Australia; 4 Neurology Department, Royal Prince Alfred Hospital, Camperdown and the University of Sydney, Sydney, NSW 2050 Australia; 5 Garvan Institute of Medical Research, Darlinghurst, NSW 2010 Australia; 6 Raphael Recanati Genetics Institute, Rabin Medical Center, Beilinson Hospital, 4941492 Petah Tikva, Israel; 7 Department of Neurology, Rabin Medical Center, Beilinson Hospital, 4941492 Petah Tikva, Israel; 8 Northcott Neuroscience Laboratory, ANZAC Research Institute, Concord, NSW 2139 Australia; 9 Molecular Medicine Laboratory, Concord Repatriation General Hospital, Concord, NSW 2139 Australia; 10 Department of Neurology, Concord Repatriation General Hospital, Concord, NSW 2139 Australia; 11 Neurodegenerative Disorders Research Pty Ltd, West Perth, WA 6005 Australia; 12 School of Medicine, The University of Western Australia, Nedlands, WA 6008 Australia

**Keywords:** *ELOVL4*, Spinocerebellar ataxia type 34 (SCA34), Erythrokeratoderma, Next-generation sequencing, Medical genetics

## Abstract

Autosomal dominant variants in *ELOVL4* cause spinocerebellar ataxia type 34 (SCA34; ATX-*ELOVL4*), classically associated with a skin condition known as erythrokeratoderma. Here, we report a large Italian-Maltese-Australian family with spinocerebellar ataxia. Notably, while there were dermatological manifestations (eczema), erythrokeratoderma was not present. Using a next-generation sequencing panel, we identified a previously reported *ELOVL4* variant, NM_022726.4: c.698C > T p.(Thr233Met). The variant was initially classified as a variant of uncertain significance; however, through segregation studies, we reclassified the variant as likely pathogenic. We next identified an individual from another family (Algerian-Maltese-Australian) with the same *ELOVL4* variant with spinocerebellar ataxia but without dermatological manifestations. We subsequently performed the first dedicated literature review of *ELOVL4*-associated ataxia to gain further insights into genotype–phenotype relationships. We identified a total of 60 reported cases of SCA34 to date. The majority had gait ataxia (88.3%), limb ataxia (76.7%), dysarthria (63.3%), and nystagmus (58.3%). Of note, skin lesions related to erythrokeratoderma were seen in a minority of cases (33.3%). Other extracerebellar manifestations included pyramidal tract signs, autonomic disturbances, retinitis pigmentosa, and cognitive impairment. For brain MRI data, cerebellar atrophy was seen in all cases (100%), whereas the hot cross bun sign (typically associated with multiple system atrophy type C) was seen in 32.4% of cases. Our family study and literature review highlight the variable phenotypic spectrum of SCA34. Importantly, it shows that erythrokeratoderma is not found in most cases and that, while a dermatological assessment may be helpful in these patients, SCA34 diagnosis should be considered irrespective of dermatological manifestations.

## Introduction

The spinocerebellar ataxias (SCAs) are a group of inherited neurodegenerative ataxic disorders characterised by progressive loss of balance and coordination. To date, at least 50 subtypes of SCA have been described [1]. Spinocerebellar ataxia type 34 (SCA34 or ATX-*ELOVL4*; OMIM #133190) is a subtype of SCA [1] first described in 1972 by Giroux and Barbeau, who reported autosomal dominant segregation of both ataxia and erythrokeratoderma variabilis (EKV) in a French-Canadian family [2]. Decades later, whole exome sequencing (WES) was performed on the same French-Canadian family, which revealed an *ELOVL4* missense variant segregating with disease in three affected individuals [3]. While SCA34 is known to be due to heterozygous *ELOVL4* variants, the pathophysiology and downstream mechanism leading to neurological dysfunction have not been fully elucidated. Additionally, SCA34 is a clinically heterogenous neurological disorder, and recent studies suggested extracerebellar manifestations of cognitive impairment, psychomotor symptoms, and retinitis pigmentosa [4, 5]. While SCA34 was prototypically described as familial ataxia with erythrokeratoderma (EK) [2, 3, 6], there have been reports of *ELOVL4* variants segregating with ataxia without EK or similar skin lesions [4, 5, 7]. In this present study, we report an Italian-Maltese-Australian family with SCA34 in the absence of EK. We also undertake the first dedicated literature review of this disorder to show that EK skin lesions are absent in most cases.

## Patients

**Fig. 1:**
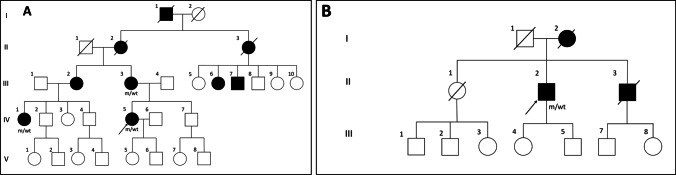
Two new families with spinocerebellar ataxia type 34 due to a heterozygous missense variant in *ELOVL4*, NM_022726.4:c.698C > T p.(Thr233Met). **A** Pedigree of the original Italian-Maltese-Australian family (family 1). **B** Pedigree of an additional family identified through PathWest Laboratory Medicine (family 2). m—*ELOVL4* variant, wt—wild type, circles—females, squares—males, filled symbol—affected, diagonal line—deceased, arrows indicate the probands

All participants provided written informed consent under protocols approved by the local research ethics committee (2019/ETH12538). The clinical phenotypes of the three affected individuals investigated in the present study are presented in Table 1. All three individuals are from the same family with an extensive history of autosomal dominant ataxia. The family is of Italian-Maltese-Australian ethnicity, and individual III-3 reports that she has several members of her extended family in Malta who are also likely to be affected by ataxia, but who were not available for clinical or genetic assessment (Fig. 1A).

**Table 1:** Clinical phenotypes of affected individuals with an *ELOVL4* variant (NM_022726.4:c.698C > T p.(Thr233Met))

	Proband (IV-5, family 1)	IV-1, family 1	III-3, family 1	Proband II.2, family 2
Ethnicity	Italian-Maltese-Australian	Italian-Maltese-Australian	Italian-Maltese-Australian	Algerian-Maltese-Australian
Age of onset	~ 20 *	~ 10 *	~ 10 *	57
Current age	48	52	72	89
Progression	Slow	Slow	Slow	Slow/progressive
Ataxia features	Gait ataxia	Gait ataxia Mild limb ataxia	Gait ataxia Limb dysmetria Mild dysarthria	Gait ataxia Limb dysmetria Mild dysarthria
Eye examination	Fundoscopy normal Horizontal nystagmus Saccadic intrusions	Fundoscopy normal. Saccadic intrusions	Gaze-evoked nystagmus Vertical nystagmus Saccadic intrusions	Fundoscopy normal. Horizontal nystagmus Intermittent pursuit Saccadic intrusions
Pyramidal tract signs	None	None	None	Brisk DTRs (2 +)
Autonomic disturbance	None	None	None	None
Cognitive impairment	None	None	None	None
Ophthalmologic findings	None	None	None	None
Dermatological findings	None (mild eczema, tinea corporis)	None	None	None
Brain MRI findings	Cerebellar atrophy Pontine atrophy	Cerebellar atrophy Pontine atrophy T2 pontine hyperintensity	Cerebellar atrophy (severe) Pontine/medullary atrophy	Pontine atrophy Cerebellar atrophy Hot cross bun sign
Other findings	No evidence of sensory loss or disturbance	No evidence of sensory loss or disturbance	No evidence of sensory loss or disturbance	No evidence of sensory loss or disturbance
SARA score	5	2	Not available	22

*The exact age of onset could not be recalled by the individuals (IV-5, IV-1, and III-3). DTRs-deep tendon reflexes, SARA-Scale for Assessment and Rating of Ataxia

**Fig. 2:**
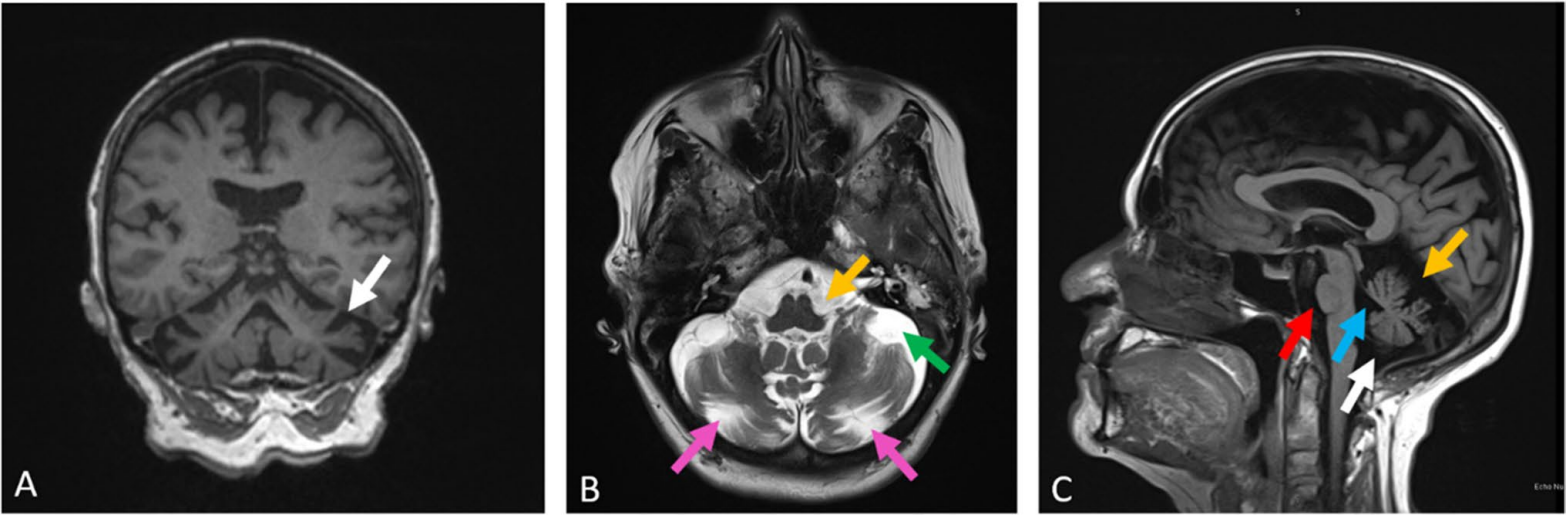
Brain MRI of III-3 showing severe cerebellar atrophy with moderate pontine and medullary atrophy. **A** T1 weighted coronal MRI showing generalised atrophy of cerebellum (white arrow); **B** T2 weighted axial MRI showing enlargement of the pontine cistern (yellow arrow), cerebellomedullary cistern (green arrow), and ex-vacuo prominence of the horizontal fissure of the cerebellum (pink arrows); **C** T1 weighted midline sagittal MRI showing enlarged pontine cistern (red arrow), fourth ventricle (cyan arrow), superior cistern (yellow arrow), and cerebellomedullary cistern (white arrow) with sparing of cerebrum

The three affected individuals presented with slowly progressive and longstanding ataxia. The age of onset could not be recalled by the individuals; however, individuals IV-1 and III-3 reported that they have had the symptoms at least since their early teenage years. They all exhibited signs of gait ataxia including broad-based unsteady gait with a negative Romberg’s test. Signs of mild limb ataxia were observed in two individuals (III-3 and IV-1), including ballistic dysmetria, dysdiadochokinesia, and heel-shin ataxia. Slight dysarthria was observed only in one individual (III-3). One individual exhibited horizontal nystagmus (IV-5), whereas another individual displayed both bidirectional gaze-evoked horizontal and vertical nystagmus (III-3). All three individuals showed saccadic intrusions into smooth pursuit eye movement. They all had normal tone, power, deep tendon reflexes (DTRs), and down-going plantar responses with no evidence of sensory neuropathy on upper and lower limb examination. Symptoms related to autonomic disturbances were not reported. Importantly, the affected individuals showed no evidence of past or current EK-related skin lesions. Of note, one individual (IV-5) was formally reviewed by a dermatologist who confirmed the lack of EK, although this patient had mild eczema of the hands and tinea corporis on the natal cleft confirmed on skin biopsy. There were no signs that indicated possible cognitive decline in any of the individuals. On brain magnetic resonance imaging (MRI), all three individuals showed cerebellar and pontine atrophy, with the older affected member (III-3) showing severe cerebellar atrophy (Fig. 2). Another individual (IV-1) also showed pontine T2 signal hyperintensity (hot cross bun sign) which is a sign typically associated with multisystem atrophy.

## Genetic Studies

The proband (IV-5) tested negative for common repeat expansions causing ataxias (SCA1, 2, 3, 6, 7, 12, 17, and Friedreich’s ataxia). Following this, the proband underwent next-generation sequencing-based custom neuromuscular gene panel (Neuro v6) testing at PathWest Laboratory Medicine. A heterozygous missense variant in the *ELOVL4* gene, NM_022726.4:c.698C > T p.(Thr233Met), was identified (rsID: rs1554162016). This result was confirmed by Sanger sequencing. This rare variant is not found in the GnomAD population database and has a high Combined Annotation Dependent Depletion (CADD) score (v1.6) of 27.5 [8]. Importantly, this variant was previously reported in SCA34 cases exhibiting typical EK lesions [6, 9, 10]. The variant was originally classified as a variant of uncertain significance (VUS) according to the American College of Medical Genetics and Genomics (ACMG) criteria (PM2, PP3, PP5) [11]. The two additional affected individuals subsequently underwent the Sanger sequencing, confirming that they carried the variant (Fig. 1A), consistent with segregation of the *ELOVL4* variant with ataxia in the family. The original VUS classification of this *ELOVL4* variant was thus upgraded to likely pathogenic (ACMG criteria: PM2, PP3, PP5, and PP1 (moderate)) [11].

## Identification of an Additional Family with the c.698C > T *ELOVL4* Variant

**Fig. 3:**
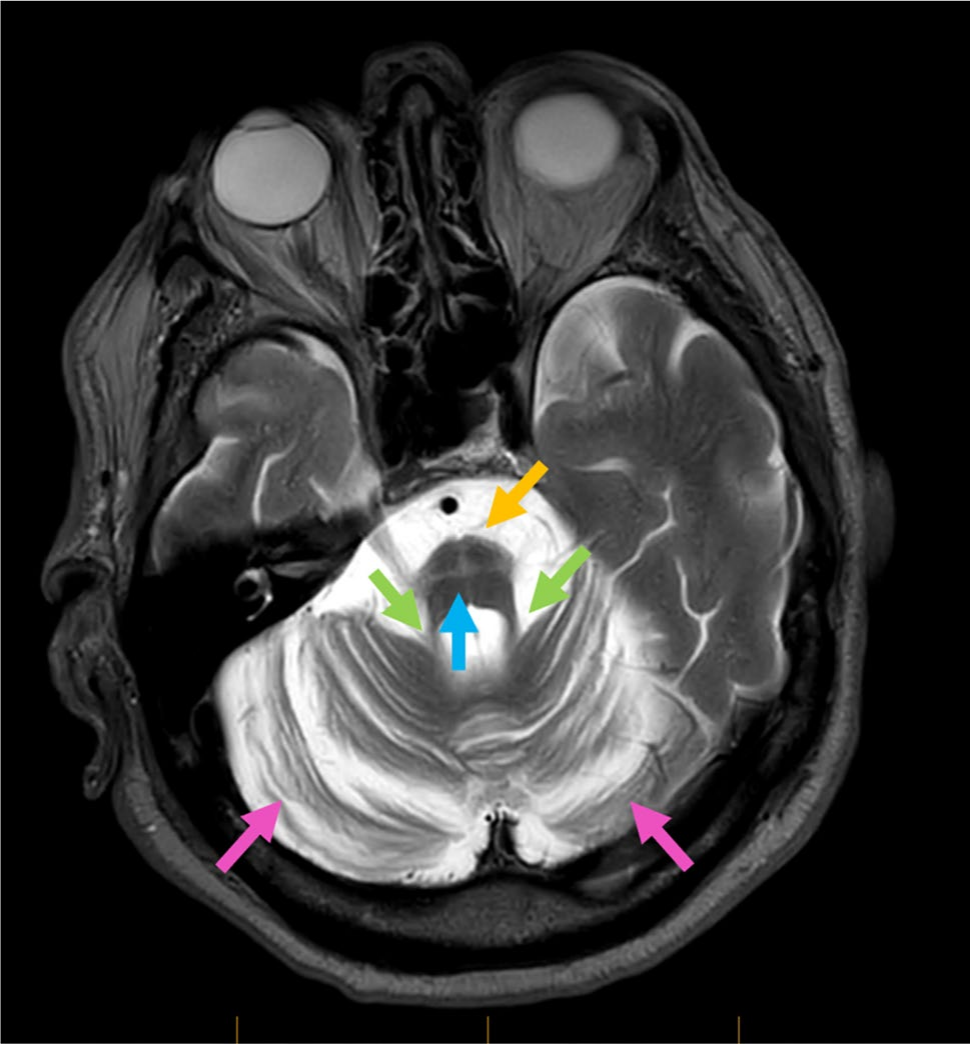
Brain MRI image of proband II showing marked atrophy of pons (yellow arrow), cerebellar peduncles (green arrows), and bilateral cerebellar hemispheres (pink arrows) with an enhanced cruciform signal on T2 and FLAIR sequence in pons, also referred to as hot cross bun sign (cyan arrow)

An additional case (individual II.2, family 2, Fig. 1B) with the NM_022726.4:c.698C > T p.(Thr233Met) heterozygous variant in *ELOVL4* was identified through PathWest Laboratory Medicine. The proband is an 89-year-old man of Algerian-Maltese origin who has a 32-year history of progressive ataxia. He now has difficulty walking and relies on a walking frame. Upon history taking, he reported intermittent diplopia although denied tremor, speech disturbance, autonomic disturbance, or cognitive decline. On examination, he had a wide-based ataxic gait which required supervision and assistance. He was unable to perform tandem gait, and the Romberg test was negative. He had mild dysarthria. He displayed nystagmus on lateral gaze, and his smooth pursuit was intermittent in both horizontal and vertical planes. Saccadic velocity and amplitude were reduced in the vertical plane but were reserved in the horizontal plane. He had normal findings on the fundoscopy assessment. On limb examination, he had brisk DTRs in both upper and lower limbs, although there was no clonus. He displayed limb dysmetria (heel-shin test) bilaterally. His brain MRI revealed marked atrophy of pons and bilateral cerebellar hemispheres with enhanced cruciform signal in the pons in T2 and FLAIR (hot cross bun sign, Fig. 3). Importantly, he did not have any dermatological findings on skin examination. Of note, his affected mother was also of Maltese heritage, but additional family members were not available to be studied. His clinical findings are summarised in Table 1 along with three individuals from the original family.

## Literature Review

A systematic literature review was performed by searching databases, including PubMed, ScienceDirect, Web of Science, and SCOPUS, using the terms “SCA34” or “ELOVL4 ataxia”. Publications with English abstracts describing cases with an identified *ELOVL4* variant were included.

In total, we identified 60 reported cases of SCA34 (Tables 2 and 3). The estimated mean age of onset was 31.85 (± 12.76) years (mean ± SD). Most cases had cerebellar ataxic features including gait ataxia (88.3%), limb ataxia (76.7%), dysarthria (63.3%), and nystagmus (58.3%). On brain MRI, cerebellar atrophy was reported in all cases (100%), and the hot cross bun sign, usually associated with multiple system atrophy cerebellar type, was seen in 32.4% of cases (Table 4).

**Table 2:** Clinical findings according to variant in reported cases of ATX-*ELOVL4*

	*ELOVL4* variant	Number of cases	Mean age of onset	Cerebellar signs			
				Gait ataxia	Limb ataxia	Dysarthria	Nystagmus
Present study	c.698C > T (p.Thr233Met)	4	24.25	4	3	2	3
Bourque et al. (2018)	c.698C > T (p.Thr233Met)	1	15	1	0	0	0
Ozaki et al. (2019)	c.698C > T (p.Thr233Met)	2	47	2	2	2	2
Wang et al. (2021)	c.698C > T (p.Thr233Met)	2	31.5	2	2	N/A	N/A
Beaudin et al. (2020)	c.504G > C (p. Leu168Phe)	9	47	9	9	9	7
Cadieux-Dion et al. (2014)	c.504G > C (p. Leu168Phe)	19	51	12	9	6	7
Bourassa et al. (2015)	c.539A > C (p. Gln180Pro)	1	25	1	1	1	1
Haeri et al. (2021)	c.539A > C (p. Gln180Pro)	1	13	1	1	1	1
Mukherjee et al. (2021)	c.539A > C (p. Gln180Pro)	1	22	1	1	1	1
Moreno-Escobar and Tripathi (2022)	c.736 T > G (p. Trp246Gly)	1	30	1	1	1	1
Ozaki et al. (2015)	c.736 T > G (p. Trp246Gly)	9	33.9	9	9	9	7
Xiao et al. (2019)	c.512 T > C (p. Ile171Thr)	10	42.6	10	8	6	5
Total		60		53	46	38	35
Mean (± SD)			31.85 (± 12.76)				
Percentage				88.3%	76.7%	63.3%	58.3%

**Table 3:** Summary of extracerebellar clinical findings in reported cases of ATX-*ELOVL4*

	Pyramidal signs	Autonomic disturbances	EK-related skin lesions	Cognitive impairment	Ophthalmologic findings
Present study	1	0	0	0	0
Bourque et al. (2018)	1	N/A	1	N/A	N/A
Ozaki et al. (2019)	2	N/A	1	1	N/A
Wang et al. (2021)	N/A	N/A	2	N/A	N/A
Beaudin et al. (2020)*	2	2	0	5	1
Cadieux-Dion et al. (2014)	7	N/A	14	0	N/A
Bourassa et al. (2015)	0	N/A	1	N/A	N/A
Haeri et al. (2021)**	0	0	0	0	0
Mukherjee et al. (2021)	1	N/A	1	1	N/A
Moreno-Escobar and Tripathi (2022)	1	1	0	N/A	N/A
Ozaki et al. (2015)	8	4	0	N/A	0
Xiao et al. (2019)	6	0	0	N/A	4
Total	29	7	20	7	5
Mean (± SD)					
Percentage	48.3%	11.7%	33.3%	11.7%	8.3%

*One individual from this study exhibited nummular dermatitis

**This case had congenital ichthyosis

**Table 4:** Summary of brain MRI finding in reported cases of ATX-*ELOVL4*

	Brain MRI findings		
	n*	Cerebellar atrophy	Hot cross bun sign
Present study	4	4	1
Bourque et al. (2018)	1	1	0
Ozaki et al. (2019)	2	2	2
Wang et al. (2021)	1	1	0
Beaudin et al. (2020)	6	6	2
Cadieux-Dion et al. (2014)	7	7	0
Bourassa et al. (2015)	1	1	0
Haeri et al. (2021)	1	1	1
Mukherjee et al. (2021)	1	1	0
Moreno-Escobar and Tripathi (2022)	1	1	1
Ozaki et al. (2015)	6	6	4
Xiao et al. (2019)	3	3	0
Total	34	34	11
Mean (± SD)			
Percentage		100.0%	32.4%

* *n* refers to the number of cases who underwent brain MRI imaging study

Importantly, EK-related skin lesions were only seen in a minority of cases (33.3%), which is in contrary to the reported prototypic manifestation of SCA34/ATX-*ELOVL4* (OMIM #133190). Whilst the majority of cases with EK skin lesions had EKV [3], several cases had lesions more consistent with progressive symmetric erythrokeratoderma (PSEK) rather than EKV [10, 12]. Of note, one study reported a 16-year-old boy with PSEK skin lesions with no neurological or cerebellar clinical signs who was found to have the c.698C > T *ELOVL4* variant [12]. This case highlights the potential clinical phenotypic variability for *ELOVL4*-associated disease, though it is plausible that this individual develops ataxia later in his life. Similarly, the aforementioned large French-Canadian family study also reported three variant carriers who displayed EKV but no ataxia which may be attributed to their younger ages [3]. Additionally, some cases demonstrated skin lesions unrelated to EK, such as ichthyosis [13] and nummular dermatitis [4], and thus were not included in the calculation of the prevalence of EK-related skin lesions (Tables 2 and 3). Interestingly, there were a total of five reported SCA34 cases with the c.698C > T variant in *ELOVL4*, and four of them exhibited EK skin lesions [6, 9, 10] in contrast to the present study where the four individuals did not exhibit EK-related skin lesions despite having the exact same nucleotide variant. This finding supports inter-familial phenotypic variability and points against a clear genotype–phenotype correlation. It may also suggest that additional factors, such as environmental and genetic modifiers, may affect the dermatological manifestation of *ELOVL4*-related disease.

In terms of other extracerebellar manifestations, pyramidal tract signs, such as DTRs abnormalities or abnormal plantar reflexes, were found in 48.3% of the cases. Autonomic disturbances, cognitive impairment, and ophthalmologic pathologies were found in 11.7%, 11.7%, and 8.3% of reported cases, respectively; however, these values may not be accurate considering the relatively small number of reports on these phenotypic manifestations. However, it should be noted that one study reported retinitis pigmentosa in four out of eight affected members in a family with c.512 T > C p.(Ile171Thr) variant [5], and another study described cognitive impairment and psychiatric symptoms in five affected members of the studied family which were characterised by c.504G > C p.(Leu168Phe) variant [4].

## Protein Modelling

**Fig. 4:**
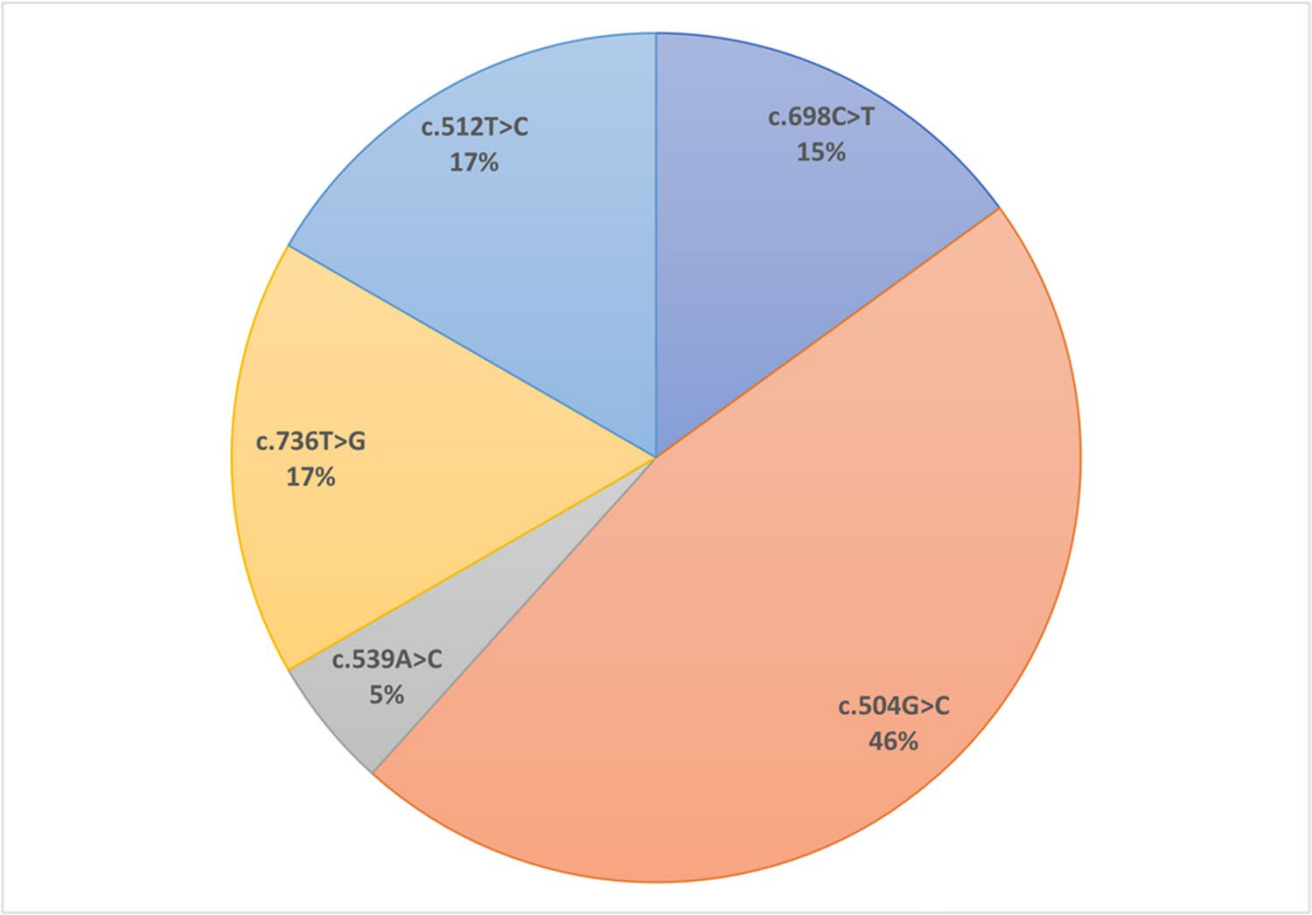
*ELOVL4* disease-causing variants and their frequency in the reported cases of SCA34

**Fig. 5:**
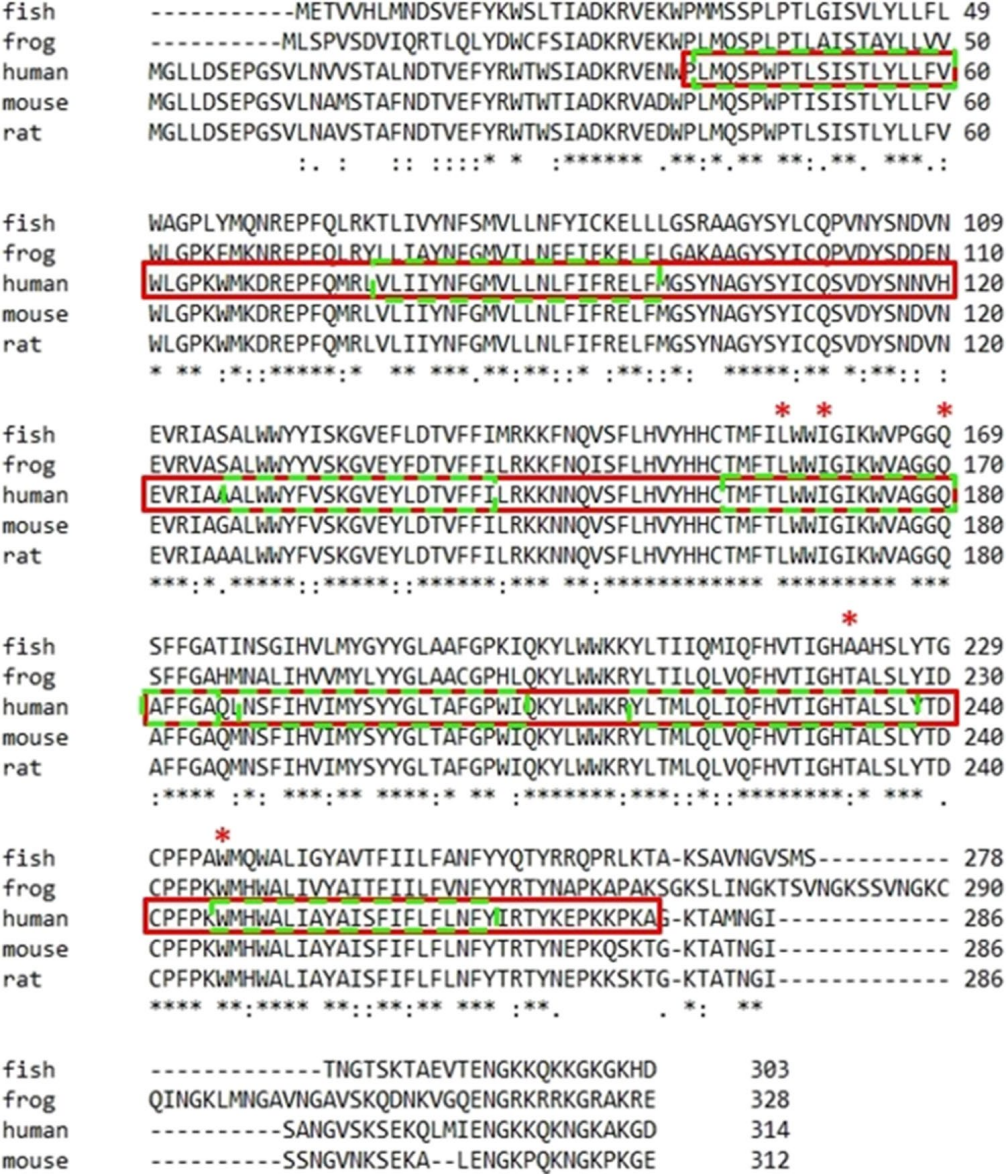
Alignment of amino acid sequences of human ELOVL4 and its ortholog in other species including mammals using ClustalW (https://www.genome.jp/tools-bin/clustalw). Red boxed region indicates conserved protein domain—ELO; GNS1/SUR4 family domain. Dotted fluorescent green box indicates the transmembrane domain. Amino acid residue positions at which variants associated with SCA34 has been reported in ELOVL4 (p.Leu168, p.Gln180, p.Trp246, p.Ile171, and p.Thr233) are indicated by an asterisk symbol (*). All the reported variants are located within the conserved protein domain and are part of the transmembrane domain of ELOVL4 protein

**Fig. 6:**
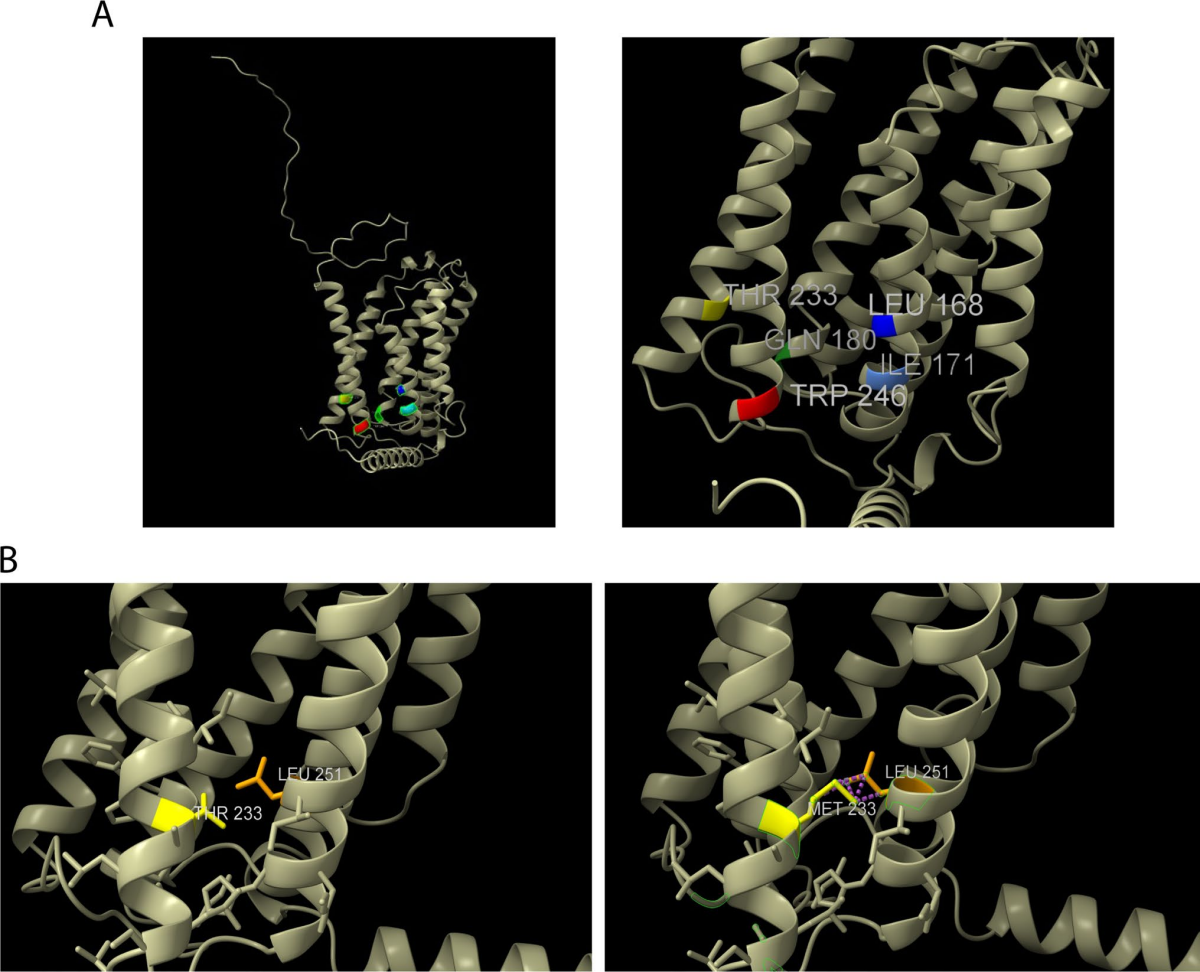
**A** (Left) Visualisation of full structure of human ELOVL4 protein using ChimeraX software. (Right) Close-up view of the highlighted residues on the left indicating SCA34 associated amino acid residues in ELOVL4. **B**. (Left) Close-up view of the p.Thr233 residue of ELOVL4 protein. (Right) ChimeraX predicted that substituting p.Thr233 residue to methionine results in disrupted interaction with amino acid residue p.Leu251 on the nearby alpha helix

Five *ELOVL4* variants associated with SCA34 (c.504G > C, c.512 T > C, c.539A > C, c.698C > T, and c.736 T > G) have been reported in the literature to date (Fig. 4). All the reported *ELOVL4* variants are located within the well-conserved region and are part of transmembrane domains of ELOVL4 protein (Fig. 5). Modelling of the predicted three-dimensional structure of ELOVL4 protein was performed using ChimeraX software [14] which revealed that missense variant c.698C>T (p.Thr233Met) may induce interference with the nearby amino acid residue p.Leu251 (Fig. 6B).

## Discussion

The present study reports segregation of a heterozygous *ELOVL4* missense variant in three affected individuals in a family with an extensive history of familial ataxia. The *ELOVL4* variant, c.698C > T p.(Thr233Met), was reclassified as a likely pathogenic variant based on segregation with the clinical ataxia phenotype within the family. An individual with spinocerebellar ataxia with the same *ELOVL4* variant from another family was subsequently identified. Of note, this individual did not have dermatological manifestations and had an affected family member (his mother) of Maltese background. We speculate on the presence of a common founder of Maltese ancestry; however, the additional individual did not wish to have further genetic studies to confirm this (an alternative explanation may be a potential hotspot unrelated to ancestry/origin).

This family study and literature review provide insights into the phenotypic heterogeneity of ATX-*ELOVL4*. Importantly, all four individuals reported in the present study displayed no evidence of EK. This was in contrast with the findings of several previous studies where SCA34 patients with the same missense variant (c.698C > T) exhibited EKV [6, 9] or PSEK [10], highlighting inter-familial variability of this disorder.

Given the inconsistent reports on EK-related cutaneous manifestations in SCA34 cases, we sought to summarise the currently reported SCA34 cases to estimate the prevalence of EK-related skin lesions (Tables 2 and 3). Here, we found that SCA34 is characterised by a variable age of onset (30.94 ± 13.71; mean ± SD) and, more importantly, only 33.3% of the currently reported cases exhibited evidence of EK-related cutaneous lesions (Table 3), suggesting a weaker correlation between *ELOVL4*-related disease and erythematous cutaneous manifestations than previously suggested.

The *ELOVL4* gene encodes a transmembrane enzyme, ELOVL4, which is involved in the elongation of very long chain fatty acids (VLCFAs). VLCFAs form part of lipid molecules which may be important for the function of the myelin sheath and cerebellum [15]. In support of this possibility, an autopsy study of a patient with established SCA34 revealed marked pontocerebellar fibre degeneration and oligodendrocyte and myelin degeneration with cerebral white matter involvement [16]. Since the myelin sheath is predominantly made of lipid molecules, it is plausible that the impaired elongation of VLCFAs, caused by *ELOVL4* variants, may impact alteration in fatty acid composition thereby suggesting a role in myelin sheath pathology. *ELOVL4* variants may directly impair oligodendrocyte function and may account for degeneration of the myelin sheath. Additionally, it has been suggested that ELOVL4 is predominantly expressed in structures which are critical in maintaining cerebellar inputs and output, such as granule cells, Purkinje cells, molecular layer interneurons, and the inferior olivary complex [17]. This predominant expression of ELOVL4 at cerebellar structures may be accountable for cerebellar pathology seen in ATX-*ELOVL4*. A recent study of a transgenic rat model of SCA34, with heterozygous c.736 T > G p.(Trp246Gly) variant, demonstrated not only that rats with the *ELOVL4* variant developed motor deficits at 2 months of age but also revealed marked reduction and depression of synapses onto Purkinje cells, the main output structure of the cerebellar cortex, through an electrophysiological study [18]. This finding further supports the essential role of ELOVL4 in cerebellar function through the maintenance of synapses at cerebellar structures. Further studies investigating the molecular mechanism of *ELOVL4* variants are required to fully elucidate the pathophysiology and to ultimately identify a therapeutic target for ATX-*ELOVL4*.

## Conclusion

To our knowledge, this is the first dedicated literature review on SCA34 (ATX-*ELOVL4*), investigating genotype–phenotype correlations. Based on the findings of the two families and our review of the published literature, we propose that the *ELOVL4*-associated disease is characterised by a variable phenotypic expression and, importantly, that SCA34 is associated with clinical ataxia phenotypes irrespective of EK skin lesions.

## Data Availability

Additional data is available on request; a data repository is not applicable.
